# Neuroactive venom compounds obtained from *Phlogiellus bundokalbo* as potential leads for neurodegenerative diseases: insights on their acetylcholinesterase and beta-secretase inhibitory activities *in vitro*

**DOI:** 10.1590/1678-9199-JVATITD-2021-0009

**Published:** 2021-06-28

**Authors:** Simon Miguel M. Lopez, Jeremey S. Aguilar, Jerene Bashia B. Fernandez, Angelic Gayle J. Lao, Mitzi Rain R. Estrella, Mark Kevin P. Devanadera, Cydee Marie V. Ramones, Aaron Joseph L. Villaraza, Leonardo A. Guevarra, Myla R. Santiago-Bautista, Librado A. Santiago

**Affiliations:** 1 Department of Biochemistry, Faculty of Pharmacy, University of Santo Tomas, Manila, Philippines, 1008.; 2 Research Center for Natural and Applied Sciences, University of Santo Tomas, Manila, Philippines, 1015.; 3 The Graduate School, University of Santo Tomas, Manila, Philippines, 1015.; 4 Institute of Chemistry, College of Science, University of the Philippines Diliman, Quezon City, Philippines, 1101.

**Keywords:** Philippine spider venom, Spider venoms, Phlogiellus bundokalbo, Neurological diseases

## Abstract

**Background:**

Spider venom is a rich cocktail of neuroactive compounds designed to prey capture and defense against predators that act on neuronal membrane proteins, in particular, acetylcholinesterases (AChE) that regulate synaptic transmission through acetylcholine (ACh) hydrolysis - an excitatory neurotransmitter - and beta-secretases (BACE) that primarily cleave amyloid precursor proteins (APP), which are, in turn, relevant in the structural integrity of neurons. The present study provides preliminary evidence on the therapeutic potential of *Phlogiellus bundokalbo* venom against neurodegenerative diseases.

**Methods:**

Spider venom was extracted by electrostimulation and fractionated by reverse-phase high-performance liquid chromatography (RP-HPLC) and characterized by matrix-assisted laser desorption ionization-time flight mass spectrometry (MALDI-TOF-MS). Neuroactivity of the whole venom was observed by a neurobehavioral response from *Terebrio molitor* larvae *in vivo* and fractions were screened for their inhibitory activities against AChE and BACE *in vitro*.

**Results:**

The whole venom from *P. bundokalbo* demonstrated neuroactivity by inducing excitatory movements from *T. molitor* for 15 min. Sixteen fractions collected produced diverse mass fragments from MALDI-TOF-MS ranging from 900-4500 Da. Eleven of sixteen fractions demonstrated AChE inhibitory activities with 14.34% (± 2.60e-4) to 62.05% (± 6.40e-5) compared with donepezil which has 86.34% (± 3.90e-5) inhibition (p > 0.05), while none of the fractions were observed to exhibit BACE inhibition. Furthermore, three potent fractions against AChE, F1, F3, and F16 displayed competitive and uncompetitive inhibitions compared to donepezil as the positive control.

**Conclusion:**

The venom of *P. bundokalbo* contains compounds that demonstrate neuroactivity and anti-AChE activities *in vitro*, which could comprise possible therapeutic leads for the development of cholinergic compounds against neurological diseases.

## Background

Spider venoms constitute a diverse and complex cocktail of compounds that are employed for defense or predatory purposes. They contain several forms of molecules such as organic compounds, linear cytolytic peptides, disulfide-rich peptides (DRPs), and enzymes that act as neurotoxic cabals and synergistically target numerous types of neuronal membrane proteins such as receptors, ion channels, transporters, and enzymes to cumulatively paralyzed their prey or predator [1]. Therefore, the diverse pharmacological targets of spider and other animal venoms have been medically used as drugs to modulate pain and other neurological conditions. To some extent, other animal venoms were also applied to treat hypertension, diabetes, blood coagulation, pain and envenomation, which are commercially available as drugs such as exenatide from gila monsters (*Helodema suspectum*), captopril from Brazilian pit vipers (*Bothrops jararaca*), bivalirudin from medicinal leechs (*Hirudo medicinalis*), ziconitide from the cone snails (*Conus magus*), fibrin sealant from South American rattlesnakes (*Crotalus durissus*), and apilic antivenom from melittin and PLA_2_ from Africanized honeybees (*Apis mellifera)* [1-9].

Moreover, there is an increasing interest in utilizing venoms to develop compounds that target neurodegenerative diseases, such as Alzheimer’s disease (AD). AD is a multifactorial neurodegenerative disorder that involves memory and cognitive deterioration in individuals leading to their incapability to carry out simple activities [10,11]. Intrinsic and extrinsic etiologies such as aberrations in brain metabolism, inflammation, genetic mutations, oxidative damage, and neurotransmitter dysfunction involving several cytosolic and membrane proteins act in a concerted and cooperative manner that contribute to the formation and aggregation of insoluble amyloid-beta (Aβ) and hyperphosphorylated microtubule binding-tau proteins into senile plaques and neurofibrillary tangles, respectively. These events primarily drive increasing cellular stress, resulting in neuronal cell death at initial brain regions, particularly the cerebral cortex, basal ganglia, thalamus, and hippocampus [12]. Afterwards, these proteins are endocytosed to other healthy neurons and subsequently drive neuronal toxicity and death. Thus, this results in loss of neurons that phenotypically leads to disrupted cognitive and motor responses from afflicted patients [13].

Additionally, one of the prominent postulations of the disease progression of AD is cholinergic hypothesis wherein the decline of an excitatory, acetylcholine (ACh), results in deterioration of cognitive and motor activities in patients [14]. ACh transmits information by interacting at post-synaptic acetylcholine receptors (AChR), which promotes downstream signaling to deliver further response to other neurons. Regulation of ACh is conducted by a post-synaptic acetylcholinesterase (AChE) to modulate synaptic transmission [15,16]. However, in neurodegenerative diseases, an increase in activities of AChE immediately decreases ACh transmission, which results in a lack of downstream signaling in post-synaptic neurons that promotes aberrant activities of beta-secretase (BACE) [17]. 

BACE is an intracellular transmembrane protease that initiates the cleavage of amyloid precursor proteins (APP) into insoluble and neurotoxic Aβ and tau proteins [17]. Aside from this, the need for elevated ACh levels is mandatory to counteract the lack of surviving neurons in neurodegenerative disease states [12,14,15]. Hence, this idea provides fundamental insights to develop AChE and BACE inhibitors to modulate disease progression [18,19]. However, the creation of safe and therapeutic BACE inhibitors is still a challenge in development, while prolonged use of AChE inhibitors might produce toxic metabolites and other side effects that can be detrimental to the patient [20,21]. 

This is the reason for developing potent, specific and non-toxic compounds that target upstream membrane proteins that are involved in normal neuronal activities to treat the disease progression [22]. Therefore, spider venom peptides, which primarily target neuronal membrane proteins to modulate the nervous system of their prey or predators, present an attractive strategy to repurpose such compounds to target relevant structural and functional equivalent membrane proteins in humans associated with neurodegenerative diseases [16,23-25].

**Figure 1.**
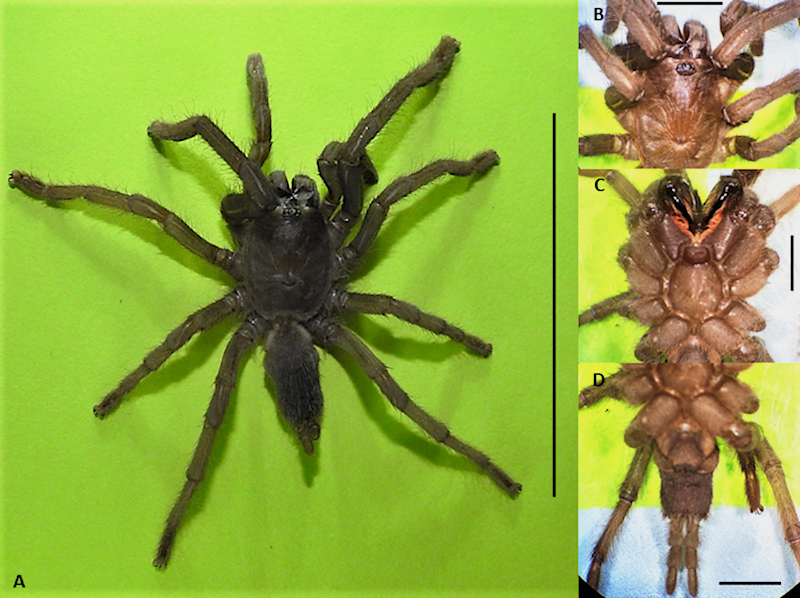
A sample of *Phlogiellus bundokalbo* that is used for venom collection and taxonomic identification. **(A)** Dorsal view, habitus, Bagacay, Surigao Island, Province of Surigao del Norte, Mindanao, Philippines. **(B)** Carapace and eyes, dorsal view. **(C)** Sternum, labium, maxilla, and coxae, ventral view. **(D)** Abdomen, ventral view. Scale bar - 30 mm for **(A)** and 5 mm for **(B)**, **(C)**, and **(D)**.

The Philippines, an archipelago of some ~7,100 islands in Southeast Asia, is teeming with diverse venomous spiders that are yet to be utilized for the discovery and development of peptide drugs against neurodegenerative diseases [26,30]. Among the endemic spiders of interest is *Phlogiellus bundokalbo* (Figure 1)*,* a tarantula belonging to Theraphosidae family first described by Barrion and Litsinger [25]. Most of the studies on this spider were limited to ecological, morphological, and taxonomical approaches [26-34]. However, research on the biological activities of its venom only focused on its cytotoxic and anti-cancer activities [35-37]. For instance, the venom of *Phlogiellus bundokalbo* exhibited cytotoxicity on human lung adenocarcinoma (A549) cells and human breast adenocarcinoma (MCF-7) cells by producing pro-oxidative radicals that impair mitochondrial membrane potential, activate caspases and initiate nuclear fragmentation [35-37]. For this reason, rigorous and extensive biomedical research is required to study the venom from *P. bundokalbo* in order to expand its utility for drug discovery and development towards neurodegenerative therapeutics. In fact, venoms from other notable species of spiders have demonstrated such activities. Two toxins from *Phoneutria nigriventer*, namely PhTx3-1 and PhTx4-5-5, were found to be neuroprotective and exhibit memory improvement in mice hippocampal neuronal slices administered with Aβ [38-40].

Moreover, PhkV, a peptide isolated from the same spider, has demonstrated antinociceptive activities through AChE inhibition which modulates the cholinergic system [41]. Aside from this, psalmotoxin (PcTx1) from *Psalmopoeus cambridgei* is currently being assessed for neuroprotective activities against neuronal acid sensing ion channels (ASICs) in preclinical studies to be suitable drug to treat hemorrhagic and ischaemic stroke [42].

Therefore, thie present study aims to evaluate the neuroactivity *in vivo* and inhibitory properties against AChE and BACE *in vitro* of fractional constituents from the venom of *P. bundokalbo*, providing an initial overview on the therapeutic potential of this venom from for application against neurodegenerative diseases.

## Methods

### Materials and equipment

All reagents and chemicals that were used in the study were analytical grade and bought from RCL Labscan, Thailand; Mym Biological Technology Company, Hi-Media Laboratories, India; Sigma-Aldrich Life Sciences; LaserBio Labs, France; Macron Fine Chemicals, Cayman Chemical Company, USA and Molecular Probes Invitrogen, USA. On the other hand, Waters Alliance e2695 RP-HPLC system equipped with w2489 UV-VIS spectrophotometer and Agilent Eclipse PlusC18 analytical column (5 µm, 5.6 x 150 mm, pore size-100 Å) were used for the fractionation of whole spider venom. Lyophilization was done using Sim International FD5-series freeze dryer. Aside from this, ThermoFisher Multiskan microplate spectrophotometer and Promega GloMax Explorer multimode microplate reader was used for the colorimetric enzymatic assays. Moreover, Shimadzu Axima Confidence Linear/Reflectron MALDI-TOF-MS system was used to determine the masses from the spider venom fractions. 

### Spider collection, rearing, maintenance and identification

**Figure 2.**
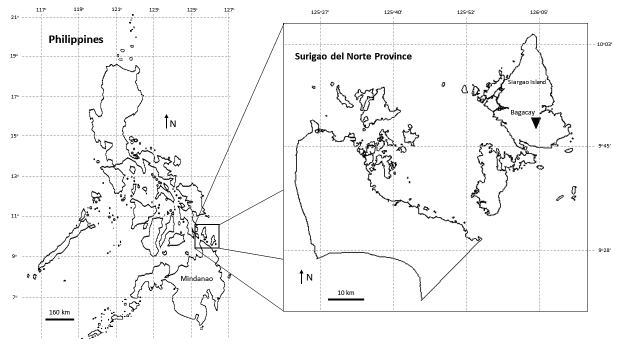
Sampling site of *Phlogiellus bundokalbo* in Bagacay, Surigao Island, Province of Surigao del Norte, Mindanao, Philippines.

The spiders were collected from the cave areas in Bagacay, Surigao, Mindanao, Philippines (Figure 2) through hand-grabbing and entrapments in their cave systems. The spiders were enclosed in 35 x 25 x 10 cm plastic boxes containing moist coconut coir beddings [43]. Then, they were maintained at room temperature with a twelve-hour light and dark cycle at the Biochemistry Laboratory of UST Central Laboratory. Afterwards, the spiders were fed with a cave dwelling cockroach *Pycnoscelsus striatus* once per week and provided with water *ad libitum* [33]. A group of the collected spiders (Figure 1) were submitted to the Museum of Natural History of the University of the Philippines Los Baños for proper identification and authentication by Dr. Aimee Lynn Barrion-Dupo through assessment of their morphological features and characteristics.

### Whole venom extraction from *Phlogiellus bundokalbo*

Spiders were initially anesthetized by carbon dioxide (CO_2_). Whole venom was collected at the chelicerae through electrostimulation at a nonlethal voltage of 35V in the microtubes and washed with 100 µL distilled H_2_O followed by centrifugation. Then, the whole venom was run in RP-HPLC, lyophilized, and stored under -20^o^ C prior to assay [43]. 

### Reverse-phase high performance liquid chromatography fractionation

Five microliters of 5.73 mg/mL of whole venom were diluted by 195 µL 0.1% trifluoroacetic acid (TFA) in distilled H_2_O in a vial with a conical insert, fractionated using C18 analytical column as stationary phase and run through a 0-95% linear gradient elution system with a flow rate of 1 mL/min for 105 min using 0.1% TFA and distilled H_2_O and 0.1% TFA in 90% acetonitrile (ACN) solvent system. The detection of the peaks was monitored at 215 and 280 nm, and the corresponding fractions were collected based on the peaks observed. Afterwards, the fractions were lyophilized and stored at -20 °C refrigerator.

### Peptide determination and quantification

Colorimetric confirmation from bicinchoninic acid (BCA) assay was employed in the whole and fractionated venom. A positive test from this assay results in a color change from colorless to deep purple. The reaction mixture contained 500 µL of BCA working reagent and 10 µL of the sample. Then, the reaction mixtures were incubated at 60 °C for 30 min and the absorbance was read at 562 nm using a UV-Vis spectrophotometer [44]. The crude whole venom sample was also quantified in BCA assay using increasing concentrations of bovine serum albumin (BSA) (R^2^ = 0.992). 

### Matrix assisted light desorption ionization-time of flight (MALDI-TOF) mass spectrometry of peptide fractions and manual curation in ArachnoServer toxin database

MALDI-TOF-MS characterization of spider venom fractions was conducted at the Institute of Chemistry, University of the Philippines Diliman. Following the standard protocol from the manufacturer for sample preparation, 1 mg/mL of spider venom fractions were dissolved in 10 mg/mL α-cyano-4-hydroxycinnamic acid (CHCA) matrix solution in the MALDI plate. Then, the plated samples were air-dried at room temperature before the sample plate was loaded and analyzed into the Shimadzu Axima Confidence Linear/Reflectron MALDI-TOF-MS system at positive ionization mode.

All spectrometric data were processed and analyzed using Shimadzu Biotech Launchpad^TM^ software, v.2.9. Spectral profiles were obtained with detection in the positive ion mass mode at a laser frequency of 50 Hz and within a mass range from 1000-4500 Da. MALDI was produced using pulsed laser light (337.1 nm, 3-ns pulse width) generated by a nitrogen laser with are petition rate of 60 Hz. Each spectrum resulted from the accumulation of data from 100 profiles, where each profile is generated from a minimum of 5 laser shots. A bias voltage of 45-80 V was applied to the sample plate. Acceleration voltage was 10 kV. External mass calibration was performed daily using the MALDI-TOF peptide mix calibration standard (757-3,657 Da) from Shimadzu. 

A database search for detected peptide masses from the venom fractions of *P. bundokalbo* was conducted by manual curation of related deposited peptides to Arachnoserver [45].

### Screening for beta-secretase inhibitory activities

The assay was performed based on the study of Je and Kim [46] and the instructions from Sigma-Aldrich Life Sciences, USA, with some modifications. The activity of BACE is dependent on the fluorescence intensity of the peptide substrate containing EDANS-DABCYL reporter molecules [H-RE(EDANS)EVNLDAEFL(DABCYL)R-OH], which are the donor and quencher, respectively.

A 10 µL of 100 µg/mL each sample (whole venom and fractionated venom) and negative control (distilled H_2_O) were added to each well of a 96-microwell plate and mixed with 90 µL of 50 mM sodium acetate buffer at pH 4.5, 2 µL of BACE substrate, and 4 µL of 1.0 U/mL of active BACE. The mixture was incubated for 60 min at 37 °C in dark. The ratiometric fluorescence at λ_em_= 495-510 nm and λ_ex_=335-355 nm of the samples was read in a fluorescence microplate reader. The negative control for the assay was 50 mM acetate buffer at pH 4.5 in the enzymatic reaction mixture. A BACE inhibitory activity graph was established to determine the percentage of BACE inhibition per venom fraction compared to the negative control [46,47]. 

### Screening and characterization for acetylcholinesterase inhibitory activities

AChE inhibition assay was adopted from the protocol of Ellman et al. [48] with modifications. The activity of AChE is dependent on the product formation of thiocholine from acetylthiocholine iodide (ATCl) which is detected by Ellman’s reagent or 5,5’-dithiobis-(2-nitrobenzoic acid) (DTNB) to form 5-thio-2-nitrobenzoic acid (TNB), a yellow-colored compound that can be detected at 412 nm using UV-Vis spectrophotometer. 

For screening of AChE inhibitory activities, 25 µL of 100 µg/mL each sample (whole venom, fractionated venom, Donepezil, distilled H_2_O) were added to each well in a 96-well microplate and mixed with 50 µL of 50 mM Tris-HCl buffer at pH 8.0, 25 µL of 1.5 mM ATCl, 125 µL of 3.0 mM DTNB and 25 µL of 0.51 U/mL AChE. In addition, the positive control used for the study was 400 µg/mL of commercial Donepezil (Aricept®), a known AChE inhibitor. The blank mixture used in the assay was composed of 50 mM Tris-HCl buffer at pH 8.9 with 3.0 mM DTNB. The microplate containing the mixture was incubated at room temperature for 5 min and read at 412 nm absorbance with 30 sec reading time intervals. Additionally, a time-dependent product formation from the enzymatic reactions was constructed to determine the inhibitory activity of the fractions with respect to the negative control, while an AChE inhibitory activity graph was created to compare the activity of venom fractions with the negative and positive controls [48,49]. 

The three fractions with potent anti-AChE activities were selected for competition experiments to determine the type of inhibition exhibited against AChE alongside the whole venom, Donepezil, and untreated AChE reactions through a concentration-dependent manner. The type of inhibition of each fraction was determined by comparing the Michaelis constant (K_M_) and maximum velocity (V_max_) values with the negative and positive controls using the kinetic graph. Lastly, an L-cysteine standard curve was established to quantify the products formed from the reactions of AChE and ATCl (R^2^ = 0.989).

### Neurophysiological evaluation of potent AChE spider venom and its fractions using *Tenebrio molitor*

Evaluation of the locomotor activity of *Tenebrio molitor* was adapted from the study of Friedel and Nentwig [50] and Hardy et al. [51] with modifications. Five microliters of 100 µg/mL sample (whole and three venom fractions) with potent activities against AChE, 400 µg/mL Donepezil (positive control) and 0.9% NSS (negative control) were used and administered between the sutures of the third metathoracic segment of each worm using Hamilton microsyringe. Rate of movements and locomotion activity were scored and observed for 15 min post-injection with 2-min interval to evaluate the effect of administration of each sample to the worm. The scoring was graded from 1 to 4, and the criteria were as follows: 4-severe, paralytic movements, insect cannot right itself; 3- continuous, twisting movements; 2- periodic, contracting movements and 1- little to no movement. A score versus time plot were established for this assay [50,51].

### Statistical analysis

Microsoft Excel version 2019, pH Stat, and GraphPad Prism 6 were used for the different statistical analyses throughout the study. Each of the assays was conducted in triplicates with varying trials for the computation of mean and standard error mean (SEM). One-way analysis of variance (ANOVA) was employed to determine the significant differences between the treated and untreated groups wherein p-values < 0.05 were considered statistically significant at 95% confidence interval. 

## Results

### Chromatographic profile and quantification of *Phlogiellus bundokalbo* whole spider venom

**Figure 3.**
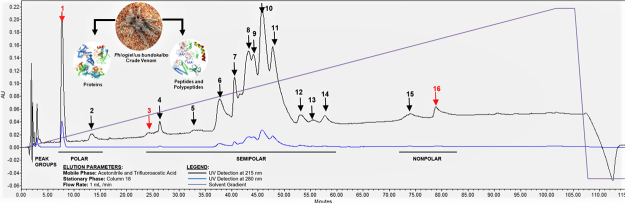
RP-HPLC profile of whole spider venom from Philippine tarantula. Arrows indicate the collected fractions with red color showing potent acetylcholinesterase inhibitory bioactivity.

The chromatogram in Figure 3 shows the whole spider venom profile which was fractionated by C18 column using ACN and TFA in dH_2_O as solvent system through gradient elution for 105 min. Also, the peaks represent different compounds detected at 215 and 280 nm predominantly indicating the presence of various forms of peptides, proteins, acylpolyamines, biogenic amines and nucleic acids, respectively. Based on the chromatographic profile, the whole venom generated 16 peaks as indicated by their retention times. The elution behavior of each peak constituent is influenced by the composition of the solvent system, ACN and TFA in H_2_O, which were semi-polar in nature and affected the elution profile of the whole venom. The peak intensities indicate differences in levels of compounds present in the venom that are principally detected in the semi-polar region.

### MALDI-TOF-MS mass distribution peptide profiles of *Phlogiellus bundokalbo* spider venom and its fractions

**Figure 4.**
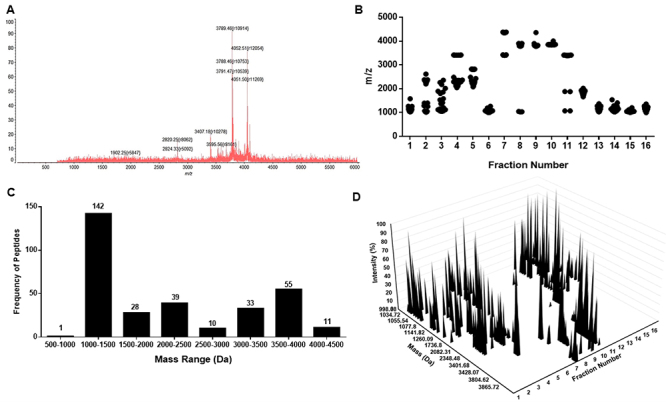
Distribution of peptide masses from *Phlogiellus bundokalbo* whole venom. **(A)** MALDI-TOF-MS spectrum of whole spider venom. **(B)** Distribution of venom peptide fractions as a function of mass-to-charge ratio collected from the RP-HPLC and compared with MALDI-TOF-MS spectra. **(C)** Histogram peptide frequency distributed and sorted according to 500 Da interval. **(C)** The 3D venom landscape of *Phlogiellus bundokalbo* venom is plotted according to the percentage area of peak intensity, mass-to-charge ratio from MALDI-TOF-MS and fraction number from RP-HPLC.

The diversity of compounds from the whole and fractionated venom from *P. bundokalbo* was explored through MALDI-TOF-MS and was measured according to their monoisotopic oxidized mass [M+H]^+^. Figure 4A shows the mass spectra of whole spider venom wherein intense signals from the peptides are detected from 900 Da - 4000 Da. Moreover, the crude sample was further fractionated and characterized by mass spectrometry to account for peptides that can be swamped out from the whole venom [52,53]. Results show that 319 peptides were detected from the fractionated whole venom ranging from 1000 Da - 4500 Da which were classified into 500 Da intervals as shown in Figure 4B and 4C Furthermore, the complexity of *P. bundokalbo* venom was plotted in a 3D landscape as shown Figure 4D wherein pronounced intensities ranging from 1000 Da - 2500 Da are observed in fractions 1-6 and 11-16, and 3000 Da - 4500 Da in fractions 7-10 [54].

### Screening for BACE inhibitory activities of *P. bundokalbo* spider venom and its fractions

**Figure 5.**
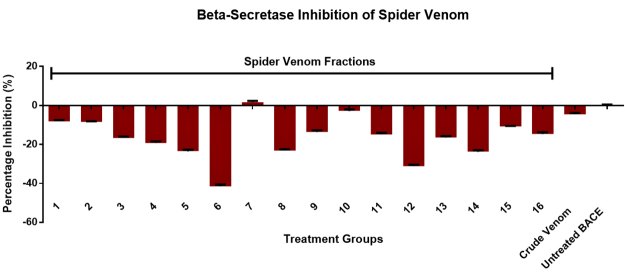
Screening for BACE inhibitory activities of whole and fractionated venom compared to untreated BACE reactions. Data is reported as mean ± SE (bars) (n = 3).

The whole and fractionated venom samples were screened for BACE inhibitory activities in comparison with the negative control or the untreated BACE reaction solution. However, most of the venom-treated samples demonstrated negative percentage inhibition which suggests that the fractional constituents of the spider venom did not exhibit BACE inhibition as indicated in Figure 5. Moreover, only F7 have shown weak inhibition to the enzyme compared to the untreated enzyme reaction and whole venom. 

### Screening and characterization of AChE inhibitory activities *in vitro* of *Phlogiellus bundokalbo* spider venom and its fractions

**Figure 6.**
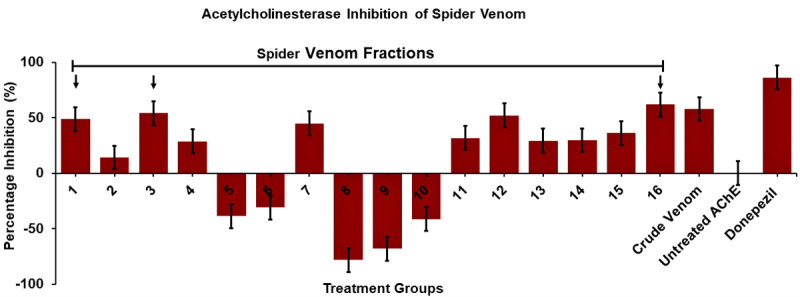
Screening for AChE inhibitory activities of whole and fractionated venom compared to untreated AChE reactions and Donepezil. Data are reported as mean ± SEM (bars). Note: *p < 0.05 significant differences against the negative control value (untreated AChE) (n = 3) (p = 0.965). Red arrows indicate the fractions with the highest AChE inhibitory activity.

**Figure 7.**
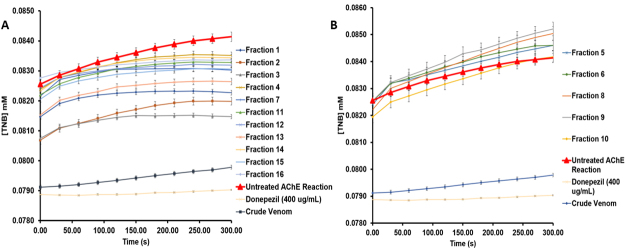
Time-dependent AChE kinetic analysis of **(A)** anti-AChE and **(B)** pro-AChE of venom fractions alongside with whole venom, Donepezil, and untreated AChE reaction. Data are reported as mean ± SEM (bars) (n = 3).

 The venom fractions were screened for AChE inhibitory activities *in vitro* compared to the whole venom, Donepezil (positive control), and untreated AChE reaction (negative control). Out of the 16 fractions, 11 of them exhibited anti-AChE activities, as demonstrated in Figure 6. Furthermore, F1, F3 and F16 showed the highest percentage of inhibition on AChE activity with values of 48.89% (± 1.31e-4), 54.33% (± 1.21e-4) and 62.05% (± 6.40e-5) respectively. In addition, the whole venom has an AChE percentage inhibition of 57.97% (± 1.99e-4), which is slightly lower than F16. In contrast, Donepezil (400 µg/mL) exhibited the highest AChE inhibitory activity of 86.34% (± 3.90e-5) compared to the fractionated and whole venom. A 5-min time-dependent kinetic graph showed in Figure 7 further validates the different responses of the whole venom, fractionated venom, and Donepezil on AChE activity. Fractions 1-4, 7, 11-16 showed an AChE kinetic curve below the kinetic curve of the negative control, while fractions 5, 6, 8 to 10 showed otherwise as represented in Figure 7A and 7B, respectively. Notably, the whole venom and Donepezil have exhibited a linear trend of AChE kinetic activity below the untreated enzyme curve which is also lower than the venom fractions indicating strong inhibition.

**Figure 8.**
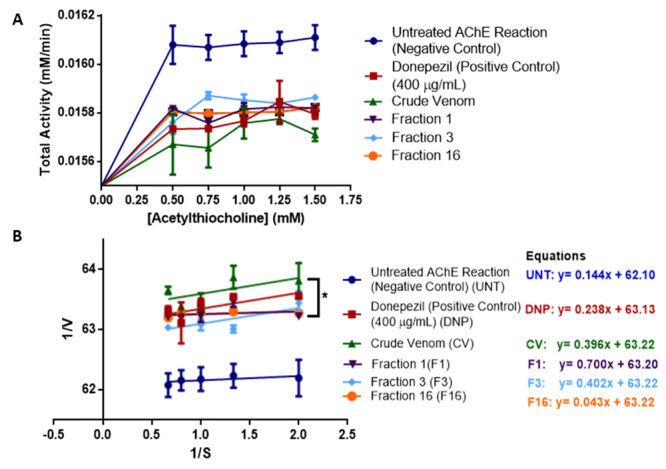
(A) Michaelis-Menten kinetics and (B) Lineweaver-Burk kinetics of venom fractions 1, 3, 16, whole venom in comparison with Donepezil and untreated AChE reaction. Data are reported as mean ± SEM (bars). Note: *p < 0.05 significant differences against the negative control value (untreated AChE reaction) (n = 3) (p = 0.001).

Moreover, F1, F3, and F16 exhibited potent anti-AChE activity which was also selected for substrate competition experiments to characterize the possible mode of inhibition and binding affinity to AChE against Donepezil and whole venom. Graphical results reported in Figures 8A and 8B showed that Donepezil, whole venom, and F3 exhibited competitive inhibition. In contrast, F1 and F16 exhibited uncompetitive inhibition as supported by K_M_ and V_max_ values derived from the Michaelis-Menten and Lineaweaver-Burke kinetics compared to the untreated AChE reactions.

The kinetic parameters Michaelis constant (K_M_) measures the affinity of substrate concentrations at ½ of the maximum velocity (V_max_) of an enzyme. The corresponding values are shown in Table 1 wherein significant kinetic inhibition was observed on the spider venom and Donepezil with respect to the untreated AChE reaction.

**Table 1.** Results of inhibition of venom fractions 1, 3, 16 and whole venom to AChE in comparison with Donepezil and untreated AChE reaction. Data is reported as mean ± SEM. Note: *p < 0.05 significant difference against the negative control value (untreated AChE reaction) (n = 3) (p = 0.0008).

Sample	K_M_ (mM)	V_max_ (mM/min)	Type of inhibition
Untreated AChE Reaction	1.15 x 10^-3^ ± 1.5 x 10^-3^	1.61 x 10^-2^ ± 3.3 x 10^-5^	-
Donepezil	4.24 x 10^-3^ ± 2.1 x 10^-3^	1.59 x 10^-2^ ± 5.6 x 10^-5^	Competitive
Whole venom	4.20 x 10^-3^ ± 4.2 x 10^-3^	1.58 x 10^-2^ ± 5.1 x 10^-5^	Competitive
Fraction 1	7.50 x 10^-4^ ± 5.0 x 10^-4^	1.58 x 10^-2^ ± 5.4 x 10^-6^	Uncompetitive
Fraction 3	4.16 x 10^-3^ ± 2.4 x 10^-3^	1.59 x 10^-2^ ± 4.3 x 10^-5^	Competitive
Fraction 16	5.60 x 10^-3^ ± 9.0 x 10^-5^	1.58 x 10^-2^ ± 7.2 x 10^-6^	Uncompetitive

### Neurophysiological evaluation via *T. molitor* locomotion of *Phlogiellus bundokalbo* whole venom and its fractions

**Figure 9.**
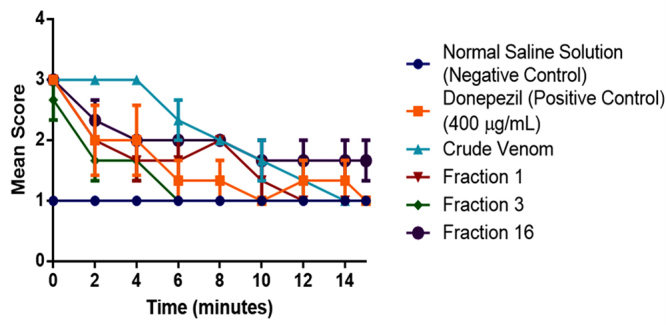
Behavioral trend observed in the locomotion of *T. molitor* after administration of Donepezil and spider venom in comparison with 0.9% NSS. Data are reported as mean ± SEM bars (n = 3).

Similarly, F1, F3, F16, whole venom, and Donepezil were administered between the sutures of the third metathoracic segment of *T. molitor* and observed their locomotory changes concerning the larvae administered with 0.9% normal saline solution (NSS) for 15 min as represented in Figure 9. Results showed that all the treated worms exhibited different levels of immediate excitation attributed to their physiological, excitatory, and motor activities upon administration of the sample.

### Manual curation of peptide masses from *P. bundokalbo* venom

Database search of the monoisotopic oxidized peptide masses of the whole and fractionated spider venom from *P. bundokalbo* showed that all the peptide masses were not similar to masses of 3 isoforms of peptides from *Phlogiellus sp.* recorded on Arachnoserver which are µ-theraphotoxin-Phlo1a (4103.79 Da), µ-theraphotoxin-Pho1b (4137.78 Da), and µ-theraphotoxin-Phlo2a (N-terminal fragment) (3276.33 Da) [45,55,56]. On the other hand, some of the peptides present in the whole and fractionated venom have similar masses present in the database from other species of spider that are related to *P. bundokalbo* by family and order as shown in Table 2.

**Table 2.** Summary of fractions from *P. bundokalbo* venom with similar monoisotopic peptide masses from related spider toxins manually curated from ArachnoServer. The first [M+H]^+^ column refers to the experimental masses detected in MALDI-TOF while the second [M+H]^+^ column indicates the matched masses in the ArachnoServer database.

Fractions	RT (min)	+ [M+H] (Da)	Matched Peptide	+ [M+H] (Da)	Species	Activity	References
6	37.72	1028.33	U -ctenitoxin-Pn1c 29	1027.57	*Phoneutria nigriventer*	Contracts smooth muscle of guinea pig ileum	Pimenta et al. [57]
7	40.555	3406.14	U -scytotoxin-Sth1a 1	3406.36	*Scytodes thoracica*	Unknown	Zobel-Thropp et al. [58]
		3410.14	κ-sparatoxin-Hv1b	3410.34	*Heteropoda venatoria*	Blocks vertebrate Kv4.x and transient outward VGKC in rat ventricular myocytes; weak blocker of calcium channels in rat cerebellar granule cells	Sanguinetti et al. [59]
		3428.07	M-zodatoxin-Lt5a	3427.94	*Lachesana tarabaevi*	Antimicrobial activity against gram-negative and positive bacteria, and yeasts. It has hemolytic activity against rabbit erythrocytes and causes non- lethal paralysis to *Musca domestica* larvae	Kozlov et al. [60]
		4356.94 4357.98	U -theraphotoxin-Cg1b 3	4357	*Chilobrachys guagxiensis*	Unknown	Chen et al. [61]
		4360	ω-hexatoxin-Hv2o	4360.05	*Hadronyche versuta*	Insecticidal toxin that blocks insect VGCC	King and Wang [62]
		4362.04	ω-hexatoxin-Hv2i	4362.03	*Hadronyche versuta*	Insecticidal toxin that blocks insect VGCC	King and Wang [62]
			ω-theraphotoxin-Bs1b	4362.23	*Brachypelma smithi*	Unknown	Escoubas et al. [63]
		4380.98	ω-theraphotoxin-Asp1a	4380.2	*Aphonopelma sp.*	Inhibits rat cerebellar granule VGCC	Nason et al. [64]
8	43.324	3792.65	U -agatoxin-Ao1d 2	3792.73	*Agelena orientalis*	Unknown	Kozlov et al. [65]
		3901.81	U -agatoxin-Ao1l 2	3901.76	*Agelena orientalis*	Unknown	Kozlov et al. [65]
9	44.143	3788.53	U -agatoxin-Ao1j 2	3788.7	*Agelena orientalis*	Unknown	Kozlov et al. [65]
		3792.56	U -agatoxin-Ao1d 2	3792.73	*Agelena orientalis*	Unknown	Kozlov et al. [65]
		3793.64	ω-hexatoxin-Ar1b	3793.51	*Atrax robustus*	Blocks insect VGCC	King et al. [66]
		3805.59	U -theraphotoxin-Hs1b 20	3805.69	*Haplopelma schimidti*	Unknown	Zhang et al. [67]
		3808.75	U -theraphotoxin-Cg1a 15	3808.73	*Chilobrachys guagxiensis*	Unknown	Chen et al. [68]
10	45.768	3849.67	μ-theraphotoxin-Hs1a	3849.66	*Haplopelma schimidti*	Blocks insect sodium channels in cockroach DUM neurons; reversibly paralyzes cockroaches and enhances the muscular contractions of rat vas deferens	Huang et al. [68]
		3850.49	U -theraphotoxin-Hhn1q 3	3850.61	*Haplopelma hainanum*	Unknown	Tang et al. [69]
		3850.97	U -theraphotoxin-Hhn1e 3	3850.61	*Haplopelma hainanum*	Unknown	Tang et al. [69]
		3862.69	U -agatoxin-Ao1s 2	3862.86	*Agelena orientalis*	Unknown	Kozlov et al. [65]
11	47.819	3404.92	U -theraphotoxin-Cv1a 1	3404.43	*Coremiocnemis valida*	Unknown	Balaji et al. [70]
Whole venom	-	3595.56	κ-sparatoxin-Hv1c	3596.46	*Heteropoda venatoria*	Blocks vertebrate Kv4.x and transient outward VGKC in rat ventricular myocytes; weak blocker of calcium channels in rat cerebellar granule cells	Sanguinetti et al. [59]
		3788.46	U -agatoxin-Ao1j 2	3788.7	*Agelena orientalis*	Unknown	Kozlov et al. [65]
			κ-theraphotoxin-Sc1a	3788.57	*Stromatopelma calceatum*	Blocks Kv4.2, Kv2.2 and Kv2.1 in vertebrates	Shiau et al. [71]
		4051.5	M-theraphotoxin-Gr1d	4051.93	*Grammostola rosea*	Antimicrobial and atrial fibrillation activity by blocking KvAP; blocks mechanosensitive ion channels	Kimura et al. [72]

## Discussion

Current therapies for treating neurological diseases are ineffective and have side effects associated with their use; therefore, there is a need to develop novel therapies. In this regard, previous studies have shown that neuroactive compounds obtained from the spider venom with neuroprotective effects *in vitro* and *in vivo* [73].

### *P. bundokalbo* venom predominantly contains peptidic compounds

The spider venom constitutes diverse mixture molecules composed of 16% of small compounds, 11% of acylpolyamines, 6% of linear peptides, 60% cysteine-knotted mini-proteins, 1% of neurotoxic proteins and 6% enzymes [1,63,74-76]. The components are primarily used for defense against predators and to paralyze their prey for ecological consumption. These toxins which include disulfide-rich peptides, acylpolyamines, and proteins affect the central and peripheral nervous system of their prey by acting on neuronal membrane proteins such as ion channels, receptors, enzymes, and transporters which cumulatively results in disruption of normal neuronal impulse, hence, phenotypically manifests as paralysis on their prey [1,74]. On the other hand, the common structural feature of spider venom peptides is cystine-knot mini-proteins. These are members of a large family of small proteins that are defined by a common structural scaffold stabilized by three intramolecular disulfide bonds [75,77]. In addition, they contain a ring formed by disulfide bridges which are the prevailing section of the peptide backbone, with a third disulfide bond penetrating the ring to generate a pseudo-knot. Furthermore, the pseudoknot provides spider venom peptides with exceptional chemical, thermal and biological stability contributing to resistance in extreme pH, organic solvents, high temperatures, and proteases [78]. In addition, prevailing motifs such as inhibitor cystine knots (ICKs) and disulfide-directed beta-hairpin (DDH) motifs are responsible for disulfide bridge formations which are highly evident in tarantula toxins [79]. Moreover, other known PTMs observed in spider venom peptides are N-terminal pyroglutamic acid additional residue, C-terminal amidation, L-D isomerization, and palmitoylation could contribute to better structural stability and activity, as well as prevention of exoprotease clevage. Aside from these, other possible PTMs that are present in other animal venoms are *N-* and *O*- linked glycosylations, hydroxylations, carboxylations, and bromination of specific amino acids. These types of PTMs alongside structurally related isoforms, variations, and molecular scaffolds, contribute to different mechanisms of pharmacologically based toxin diversifications in spider venom [80-82]. 

In *Phlogiellus bundokalbo* venom, the chromatographic profile contains peaks detected at 215 nm and 280 nm which generally detect peptides as indicated in Figure 3. Based on the UV spectra library, the wavelength range for the detection of peptide bonds is at 210-220 nm, while for aromatic groups, phenylalanine (F), tyrosine (Y) and tryptophan (W) is detected at 280 nm. Aside from this, the identities of each fraction can be deduced spectrophotometrically wherein the peaks with highest intensity mostly contain peptides and proteins as compared with peaks with lower intensities [83]. 

In a similar way, analysis of the chromatogram suggests that the compounds present on peaks 1 and 2 are characterized to be polar in nature, peak 3 to 14 to be semi-polar in nature, while peaks 15 and 16 are characterized to be non-polar due to their affinity with the solvent system. Likewise, peaks 2-16 exhibited broad peaks which can be attributed to the solvent conditions established in the RP-HPLC or to the nature of the fractions themselves, which may contain different compounds in a single peak. Moreover, multiple peaks from 5-14 may indicate similar characteristics of each compound present in the chromatogram with respect to their amphiphilicity. The elution of each peak is influence by the nature of the solvent system wherein TFA and ACN increases the resolution of the peaks in the chromatogram by acting as counter ions to the silanol groups in the stationary phase and increase the hydrophobicity of the mobile phase, respectively, to prevent the adsorption of peptides in the column [84,85]. 

However, these wavelength ranges are not specific only for peptide bonds and aromatic amino acids as they can be detected for small molecules such as acylpolyamines, organic acids, nucleotides, and free amino acids which are also present in the whole spider venom [74,83]. Furthermore, spider venom contains various forms of proteins and enzymes such as phospholipases, hyaluronidases, phosphatases, esterases, and proteases, all of which act as toxins and necrotic factors that are responsible for the damage and lethality at the superficial and deep parts and organs of their predator and prey. Moreover, early eluting peaks or polar compounds in RP-HPLC of various tarantula venoms indicate the characteristic of low molecular weight compounds (n<2000 Da) such as biogenic amines, polyamines, and small peptides. Also, compounds at 35-55% of ACN may indicate the presence of peptides with molecular masses at 2000-8000 Da range, while late eluting compounds may represent high molecular weight compounds with masses greater than 10,000 Da [86]. In contrast, MALDI-TOF-MS analysis of *P. bundokalbo* fractions indicates that early eluting fractions, F1 and F2, contain peptidic compounds that are less than 2000 Da. Moreover, semipolar eluting fractions, F3 - F14, have peptidic constituents with mass ranges of 2000 Da-4500 Da, and late eluting fractions, F15-F16 contain peptide masses ranging from 1000-2000 Da. These can be attributed to the nature of the sequence, folding, functional groups, and PTMs, which may confer different interactions between constituents in the whole spider venom and their corresponding retention time to the stationary phase [87].

Finally, manual curation of peptides from *P. bundokalbo* in the Arachnoserver, shows that most of the peptide masses from the venom no similarity to the available *Phlogiellus sp.* toxins in the database. Moreover, it is interesting to note that some of the fractions which demonstrated either anti-AChE or anti-BACE activities have shown similarity of peptide masses to other spider toxins that are related to *P. bundokalbo* by family *Theraphosidae* and order *Araneae* suggesting that the peptides from *P. bundokalbo* have not yet recorded in the database [45].

### BACE proteolytic activity influences loss of activity on *P. bundokalbo* venom peptides *in vitro*

BACE is a dimeric and transmembrane aspartic endoprotease localized in the Golgi apparatus of neurons that cleaves several neuronal peptide substrates, including amyloid-precursor protein (APP) [17]. It attaches to the ER and Golgi membrane through its transmembrane and cytoplasmic domain at its C-terminus, which determines its structure, retention, and enzymatic activity [88]. Intrinsic and extrinsic perturbations such as genetic mutations, cellular stress, modifications, inflammation, and other signaling anomalies in neurons contribute to alterations in biosynthesis and activity of BACE, which includes a residue-specific cleavage of APP that leads to the formation of insoluble amyloid-beta (Aβ) or insoluble senile plaques [17,89]. The aggregation of insoluble Aβ is one of the main effectors that exacerbates neuronal toxicity, which includes disrupting cellular membranes to form membrane-protein like structures to allow the influx of ions that impair normal cellular processes and initiates mitochondrial oxidative and endoplasmic reticular stress that continually generates toxic proteins and ultimately results to neuronal cell death [13]. Hence, this leads to less neurons transmitting stimuli and responses that phenotypically disrupt cognitive and motor function in patients [90]. Thus, several peptides, antibodies, and small molecule BACE inhibitors were developed primarily from natural products to generate therapeutic leads against BACE [91].

However, most fractional constituents of the spider venom did not exhibit inhibition towards BACE, possibly because the enzyme has numerous peptide substrates where BACE cleavage may occur depending on the degree of activity [47]. Since the spider venom primarily contains peptides as characterized by UV wavelength, colorimetric determination, and MALDI-TOF-MS, cleavage of venom peptides by BACE may result in loss of activity towards its inhibitory activity. Aside from this, BACE is active at acidic pH ranging from 4.0-5.0, which linearizes and proteolyzes the spider venom peptides by reduction of their distinct PTM, disulfide bridges (S-S) to cysteine (S-H) groups because the -SH functional side group of cysteine has a pka of 8.8 [92]. Thus, it is hypothesized that pka values lower than 8.8, such as the case of the acidic pH environment of BACE, protonates the cystine disulfide bridges of spider venom into cysteine (S-H) allowing BACE proteolytic cleavage which leads to loss of activity of the peptides [92]. Moreover, the regions of the enzyme explained through several works may provide structural insights wherein the venom peptides may contain intrinsic properties towards enzyme activation instead of inhibition [88,93]. Finally, there were no related reports that establish spider venom peptides' intrinsic activity towards BACE inhibition *in vitro*. 

### *P. bundokalbo* venom peptides demonstrates neuroactivity and anti-acetylcholinesterase impact

AChE (E.C. 3.1.1.7) is a 550 amino acid residue carboxylesterase monomer that hydrolyzes acetylcholine into its choline and acetate derivatives [94]. It localizes in the membrane of the post-synaptic neuron by assembling into tetramers through its tryptophan amphiphilic tetramerization (WAT) domain helices located in the C-terminus region AChE monomer into the proline-rich attachment domain (PRAD) of the proline-rich membrane anchor (PRIMA) protein to form [AChE^4^-PRIMA] complex [95]. Subsequently, three molecules of [AChE^4^-PRIMA] complex attaches to the collagen Q inside the cell through the cytoplasmic domain of PRIMA protein to “lock” the attached complex and finally form [AChE^4^-PRIMA]_3_COLQ super complex [96,97]. The assembly of multiple AChE monomers at the post-synaptic neuron membrane facilitates rapid turnover of acetylcholine after binding and eliciting signals to acetylcholine neurons to regulate the cognitive and motor activities of an individual [94]. 

However, according to the cholinergic hypothesis in AD, excessive alterations in AChE alongside reduced cortical innervations, corticocortical glutamatergic neurotransmission, coupling of muscarinic M1 receptors to secondary messengers inside the post-synaptic neuron are contributory factors towards the aggravation of tau protein formation, amyloidogenesis, and amyloidosis which are symptomatic hallmarks of AD [14]. Thus, cholinergic hypothesis provided the foundations for neurotransmitter-based drugs such as acetylcholinesterase inhibitors, most notably, Donepezil. In a neurodegenerative disease-state in patients, the dwindling number of surviving neurons necessitates high amounts of neurotransmitters, in this case, acetylcholine to continue the normal cognitive and motor activities. Therefore, to carry out this event, AChE should be inhibited by donepezil to temporarily increase the number of ACh levels in neurons and facilitate neuronal transmission [18]. 

Based on the screening results for AChE inhibitory activities, the spider venom generally exhibited weak inhibition in comparison with Donepezil, as indicated in Figure 6. These can be attributed to several factors affecting the weak inhibition of the spider venom *in vitro* against AChE, which are the diversity of compounds and concentrations of each fraction, the molecular size of the compound present in each fraction, and the nature of the spider venom fractions with respect to their amino acid compositions, functional groups and PTMs towards AChE inhibition.

First, the presence of various compounds in each fraction influences the weak inhibition of AChE which is manifested in the broad and multiple peaks in the chromatogram and the mass distribution profiles in MALDI-TOF-MS. Most notably, this is evident in peaks 6-11 of the chromatogram in Figure 3 of the spider venom, suggesting that there are many compounds present on their similarity in polarity [98]. Another thing to consider is the size and nature of the compounds present in each fraction which affected AChE inhibition *in vitro*. Since spider venom contains different sizes of post-translationally modified peptides and polypeptides with molecular weight (MW) ranges greater than 2,000 Da, and are more significant than Donepezil, a small molecule with M2 of 379.42 Da, it is unlikely that the binding sites for AChE inhibition are the same for the spider venom and donepezil, thus, contributing to weak inhibition [81,82]. Small molecules can burrow deep into the active site of AChE to exhibit potent inhibition unlike peptides, polypeptides, and mini proteins that can only interact at the periphery of the active or allosteric site on AChE due to their size, thus, may explain the weak inhibition. Likewise, the possible covalent and noncovalent interactions such as disulfide linkages, hydrophobic interactions, van der Waals, London dispersion forces, hydrogen and ionic bonding of spider venom components, and Donepezil via their functional groups to the enzyme are contributory factors to the degree of AChE inhibition [99]. 

Furthermore, this can be supported by the kinetic inhibition characterization as indicated in Figures 7 and Table 1, which may explain the possible molecular interactions between spider venom peptides, Donepezil, and AChE to exhibit corresponding competitive and uncompetitive inhibitions. First, for competitive inhibitors, which are Donepezil, whole venom, and F3, they bind at the active site of AChE which is the same binding site for ACh for substrate hydrolysis. This could be accredited to the small peptides with MW of less than 2000 Da or molecules present in the whole venom and F3, which manifest weak competitive inhibition compared to Donepezil, which presents strong competitive inhibition in comparison with the untreated AChE reaction. It can be inferred based on related literatures that the mechanism of inhibition of competitive inhibitors can be attributed to their direct interaction at the catalytic triad, peripheral anionic site, oxyanionic site and choline binding site of AChE to prevent substrate hydrolysis and thereby lower the kinetics of AChE [94]. In addition, it can also be attributed to certain functional groups present especially on the spider venom such as keto groups, carboxyl groups and methoxy groups which interact at the hydroxyl groups of Ser203 in the catalytic triad of AChE to prevent further modification and hydrolysis of ACh [94,100]. Furthermore, hydrophobic and positively charged or N-containing functional groups may influence conformational change-induced enzyme inhibition to prevent ACh hydrolysis at the catalytic site of AChE by interaction at specific amino acid residues such as Gly118, Gly119, Ala201 present in the anionic site of the enzyme [94,95]. In the same way, compounds in venom fractions 1 and 16 that exhibited uncompetitive inhibition to AChE-ACh complex *in vitro* can be attributed to the binding interactions of the compounds at the peripheral anionic sites nearby the choline-binding pocket of the active site, which contains positively charged groups such as amino groups and other N-containing compounds [94]. Aside from this, allosteric inhibition may also be one of the factors for inhibition of AChE because venom peptides may also interact at other sites of AChE, which can induce a conformational change to the structure of AChE and can influence ACh hydrolysis inhibition [94,101]. On the contrary, fractions that show negative inhibition against AChE suggest that they may increase the enzyme activity by interacting in allosteric sites that induce enzyme activation instead of inhibition which may not be ideal if the compounds present in these fractions are developed into AChE inhibitors [102,103]. On the other hand, a study from Undheim et al. [104] that the venom of a related theraphosid spider, *Trittame loki*, contains AChE as part of its arsenal. These may also explain the negative inhibition because the additional AChE from *P. bundokalbo* venom may promote ACh catalysis than inhibiting it [104]. Nonetheless, these also suggest that they can be repurposed to other cholinergic-related targets that induce activation or addition of AChE instead of inhibiting it such as in the case of cholinergic poisoning wherein recombinant cholinesterases can be used to rapidly hydrolyze nerve poisons to prevent paralysis in a patient [105].

Aside from this, behavioral results from *T. molitor* principally contains high levels of neurotransmitters for chemo- and mechanosensory receptors neurons in the peripheral nervous system (PNS) and central nervous system (CNS) of insects. Interestingly, the nervous system of the *T. molitor* is comprised of several receptors such as acetylcholine, GABA, and glutamate receptors to mediate the regulation of synaptic transmission [74]. Roughly, the nervous system of *T. molitor* is comparable with that of a human because these insects have functional equivalents of neurotransmitters and neuronal membrane proteins in the body, which are relevant to study for neurodegenerative diseases [106]. Thus, this provides the foundation for *T. molitor* as an organism to study the neuroactivity effects upon administration of Donepezil and spider venom *in vivo*. The scoring was based on the rate of motility and convulsive/paralytic-like effects of the venom and Donepezil towards the larvae, wherein 4 is the highest while 1 is the lowest. This is highly attributed to the *in vivo* action of the venom fractions, whole venom, and Donepezil on *T. molitor,* wherein spider venom toxins may cause a build-up of neurotransmitters at the synapse which may interact at the pre-synaptic neuronal receptors for a prolonged amount of time in the nervous system of the *T. molitor*, and phenotypically results to excitations and increase motor activities [50,51,55]. Based on the results from Figure 9, all the mealworms administered with Donepezil whole and selected venom fractions exhibited intense quivering, jerking forward, and backward locomotor activities which can be attributed to excitatory-like effects due to build-up and increased in the concentration of several excitatory neurotransmitters in the synapse of the insect nervous system to transmit elevated neuronal signals in their body. This neuroactivity towards membrane proteins is also shared with other venom toxins from scorpions, snails, snake, and other insects and arthropods which interact with nAChR, mAChR, adrenergic transporters and receptors, glutamatergic receptors, transient receptor potential (TRP) channels, and voltage-gated sodium, potassium and calcium channels which affect neurotransmitter release, recognition, signaling, and uptake [41,107,108]. The results from the behavioral evaluation of *T. molitor* and AChE inhibition studies suggest that the fractional constituents from *P. bundokalbo* venom are insightful compound ds to focus on cholinergic therapeutics with respect to neurological diseases [109]. Through understanding the complex picture of cholinergic transmission, other receptors, ion channels, and transporters (e.g., GABA receptor, glutamate receptors, voltage-gated sodium, potassium, and calcium channels, and acetylcholine transporters) are also involved in delivering neuronal information which could also be affected in neurodegenerative disease state as demonstrated by cholinergic hypothesis in AD [12,14,15,110]. However, since the etiology of neurodegenerative diseases are multifactorial that mainly involves signaling through membrane proteins and the pharmacological activities of the spider venom compounds that target membrane proteins, this shows that they can also be studied to modulate other membrane proteins, aside from AChE, with the focus on discovery and development of cholinergic-related compounds. 

## Conclusion

In this preliminary study, the *P. bundokalbo* spider venom fractions from Mindanao, Philippines, contain compounds that showed notable behavioral activities *in vivo* and AChE inhibitory activities but not in BACE *in vitro*. Hence, further research on *P. bundokalbo* venom should focus on developing it for cholinergic studies such as investigating cholinergic-related membrane proteins (e.g., ion channels, receptors, and transporters) aside from AChE that contributes towards plaque and tangle degradation, modulation of neuroinflammation, and neuroprotective effects against Alzheimer’s disease and other neurological diseases. Similarly, dose-dependent evaluation, purification, identification, and structural elucidation of putative cholinergic compounds from the spider venom are essential to developing into its more potent, stable, and target-specific forms against neurodegenerative diseases. 
